# Evaluation of Sterify Gel as an Adjunctive Treatment to Scaling and Root Planing in Promoting Healing of Periodontal Pockets: A Split-Mouth Randomized Controlled Trial

**DOI:** 10.1155/2024/3113479

**Published:** 2024-01-04

**Authors:** Luca Levrini, Michela Rossini, Elisa Truppello, Simone Sevi, Enrico Fiorini, Stefano Benedicenti, Claudio Pasquale, Davide Farronato

**Affiliations:** ^1^Department of Human Sciences, Innovation and Territory, Postgraduate School of Orthodontic, University of Insubria, 21100, Varese, Italy; ^2^Department of Medicine and Surgery, School of Dental Hygiene, University of Insubria, 21100, Varese, Italy; ^3^Department of Surgical and Diagnostic Sciences, University of Genoa, 16132, Genoa, Italy

## Abstract

**Background:**

Periodontal disease is a common infectious disease that leads to the destruction of tooth-supporting structures. Current treatments, such as scaling and root planing (SRP), have limitations in deep and complex pockets, and antibiotic use carries the risk of resistance. Sterify Gel, a medical device composed of polyvinyl polymers, hydroxytyrosol, nisin, and magnesium ascorbyl phosphate, offers a new approach to periodontal care. This study aims to evaluate the safety and efficacy of Sterify Gel as an adjunctive treatment to SRP in promoting the healing of periodontal pockets.

**Methods:**

The study includes 34 patients with moderate to advanced chronic periodontal disease. Randomization assigned one site for SRP alone (control) and the other site for SRP with Sterify Gel (treatment). Periodontal parameters were evaluated at baseline, 1, 2, and 3 months after treatment bacterial contamination was assessed through quantitative PCR at baseline and 3 months after treatment. Statistical analysis was conducted using ANOVA and Wilcoxon test.

**Results:**

Treatment with Sterify Gel and SRP demonstrated significant improvements in pocket depth, gingival recession, and clinical attachment level compared with SRP alone. Bleeding and plaque indexes, pain perception, tooth mobility, and furcations showed no significant differences between the two groups. The treatment group showed a reduction in bacterial contamination at 3 months.

**Conclusions:**

Sterify Gel in combination with SRP shows the potential for improving periodontal health by promoting healing and reducing periodontal pockets. It may offer benefits in preventing bacterial recolonization and reducing reliance on antibiotics.

## 1. Introduction

Periodontal disease is a disease of infectious origin affecting about 70% of the adult population that, as a result of the chain of inflammatory reactions triggered, leads to the destruction of the supporting apparatus of the tooth. The complex bacterial biofilm that colonizes tooth surfaces underlies the infection that starts as gingival inflammation and then extends to the periodontal ligament and alveolar bone [1]. In clinical terms, we observe a “periodontal pocket,” a condition where the gum tissue is detached from the tooth, causing it to rest against the tooth without proper adhesion. The microenvironment of the pocket is ideal for promoting the proliferation of aggressive bacteria that can perpetuate the damage over time. Measurement of the periodontal pocket through a probe, detection of the bleeding index, and, in some cases, bacterial typing with DNA probes are the primary diagnostic indicators for the severity of the pathology [2, 3]. In a healthy periodontium, there is no detachment of the gingival epithelium from the tooth, the space between the two is no less than 2 mm deep, and the bleeding index is almost zero [4].

Available treatments include nonsurgical therapy from the outset, which includes sessions of professional dental cleaning using ultrasound and airflow with glycine or erythritol to break down supragingival bacterial biofilm and decontamination of periodontal pockets through root surface debridement (RSD) and scaling and root planing (SRP) that remove subgingival plaque and tartar. The aim is to mechanically remove the causative factor of periodontitis and thus promote natural healing of the gingiva and reduction of periodontal pockets [5]. However, both the depth and anatomical complexity of the pocket sometimes limit such maneuvers, and based on this, new bacterial colonization can infiltrate and reinfect the pockets after RSD and SRP, inhibiting the healing process [6].

Adjunctive treatments combined with RSD and SRP have been developed to improve gingival healing and prevent recurrent infection and inflammation of periodontal pockets. Some of these methods include antimicrobial laser therapy through the activation of a photosensitive molecule (photodynamic therapy) [7], or the use of antibiotics or antimicrobial molecules carried directly into periodontal pockets to reduce the bacterial load present and prevent reinfection. Release can be achieved by using paste or scaffolds, which are placed in pockets to allow for local and gradual distribution of antimicrobials over a period of days or weeks [8]. In addition to local delivery, antibiotics can also be administered systemically [9]; however, there is a risk of causing antibiotic resistance, which, in addition to being a significant health risk to the world's population [10, 11], can worsen the patient's clinical picture by rendering standard antibiotic treatments ineffective [12].

Other treatments involve the reduction of pocket depth (PD) by resective or regenerative techniques through surgical approaches to restore the system to health. Although numerous clinical trials demonstrated the efficacy of SRP, treatments in combination with this technique can dramatically improve clinical parameters and prevent recurrence [13, 14].

In the search for alternative treatments that do not rely on antibiotics, Sterify Gel has been developed as a potential solution. The design of this Class III medical device addresses the challenges associated with periodontal pockets, offering a new approach in the field of periodontal care. Composed of polyvinyl polymers, hydroxytyrosol (HT), nisin, magnesium, ascorbyl phosphate (MAP), and citrate buffered saline, Sterify Gel presents unique properties that contribute to its potential efficacy. With its viscoelastic and mucoadhesive characteristics, the gel can effectively penetrate even the most challenging and inaccessible areas within the periodontal and peri-implant pockets, similar to other gels' behavior [15, 16]. The inclusion of HT as an antioxidant [17, 18] and nisin as a preservative bacteriostatic agent [19] adds further stability to the gel formulation [20]. In the context of scientific exploration, Sterify Gel represents a noteworthy avenue for investigation as a potential antibiotic-free adjunctive treatment for periodontal pockets.

This randomized controlled trial aims to evaluate the safety and efficacy of Sterify Gel as an adjunctive treatment to SRP in promoting healing of periodontal pockets. The study aims to compare the efficacy of SRP alone with SRP combined with Sterify Gel, which may find useful application in cases of moderate to advanced chronic periodontal disease. The trial's objective is to improve and accelerate healing parameters and prevent the recurrence of inflammation and infection. In addition, Sterify Gel's mechanism of action may reduce the use of antibiotics and the associated risk of antibiotic resistance, allowing for the maintenance of patients who cannot undergo surgical treatment.

## 2. Materials and Methods

### 2.1. Selection Criteria

The study includes patients with Grades III and IV chronic periodontal disease according to the European Federation of Periodontology classification involving at least two sites. Sites needed to be distant and in different quadrants. Inclusion criteria include an age older than 18, a minimum of six teeth with a periodontal PD of more than 5 mm, and at least 20 teeth. Exclusion criteria include hypersensitivity to one or more components of the device, pregnancy or breastfeeding, heavy smoking, concomitant dental disease, diabetes mellitus, rheumatoid arthritis, aggressive periodontitis, history of radio—or chemotherapy, mucosal autoimmune disorders, mental illnesses, parafunctions, such as bruxism, use of antibiotics in the last 3 months, and periodontal surgery in the previous 12 months in the study areas. The study population comprises 34 subjects for a total of 68 treated sites.

### 2.2. Study Design

The study was a prospective, randomized, controlled, and split-mouth investigation study designed to assess the safety and efficacy of Sterify Gel (Sterify, Turin, Italy) as an adjunctive treatment following nonsurgical SRP for periodontitis in comparison with SRP alone.

### 2.3. Medical Device Features

Sterify Gel is a CE-certified (CE 0426) sterile ready-to-use hydrogel mainly composed of polyvinyl alcohol and polyvinylpyrrolidone (PVP) in citrate buffer solution, with the addition of three excipients, namely hydroxytyrosol (HT, visco-modulator), magnesium ascorbyl phosphate (MAP, gamma ray protector), and nisin (preservative).

### 2.4. Study Procedures

Following informed consent, each patient's mouth was divided into segments to identify two or more sites to be treated. Simple randomization was used to assign one site the nonsurgical procedure only (control) and the other site the nonsurgical procedure associated with the use of Sterify Gel (treatment). This was executed using a virtual coin flip to ensure 50/50 probability of site assignment to each group. The nonsurgical procedure involved SRP under local anesthesia with EMS scalers and Gracey curettes (Hu-Friedy, Chicago, Illinois, United States). After the procedure, Sterify Gel was applied directly inside the periodontal pocket in the treatment group with the help of a 23G blunt tip needle. Volume of injection was up to 0.3 ml. Participants followed a standard oral hygiene program, which included twice-daily brushing with fluoride toothpaste and daily use of dental floss or interdental brush, except on the day of treatment when patients were advised not to clean the interdental spaces. Data collection included periodontal PD, bleeding index, plaque index, gingival level of recession, level of clinical attachment, degree of mobility, furcations, and pain, which were evaluated at baseline, 1, 2, and 3 months after treatment. Examiners in this study were not blinded to the treatment assignments of the sites. Detection and quantification of periodontal bacterial contamination were assessed through periodontitis DNA test based on semiquantitative qPCR technique (LabOral Diagnostics, Houten, Netherlands) at baseline and 3 months after treatment. Samples were collected by gently inserting the sterile probes (provided within the test kit) into the periodontal pockets for a few seconds.

### 2.5. Data Analysis

The authors used Graph Prism 9 to analyze the data. The differences in evaluation parameters recorded at different time points between the control and treatment groups were analyzed using ANOVA both for intratreatment differences in the different timings per treatment and intertreatment differences between the different treatments at the same timings. Intertreatment differences were also tested with nonparametric Wilcoxon test.

### 2.6. Ethics

The study received approval from the *Comitato Etico dell'Insubria* on 9 August 2022 and the Italian Ministry of Health on 2 December 2022.

## 3. Results

### 3.1. Changes in Pocket Depth, Gingival Recession, and Clinical Attachment Level after Treatment with Sterify Gel Compared to SRP Alone

**Figure 1:**
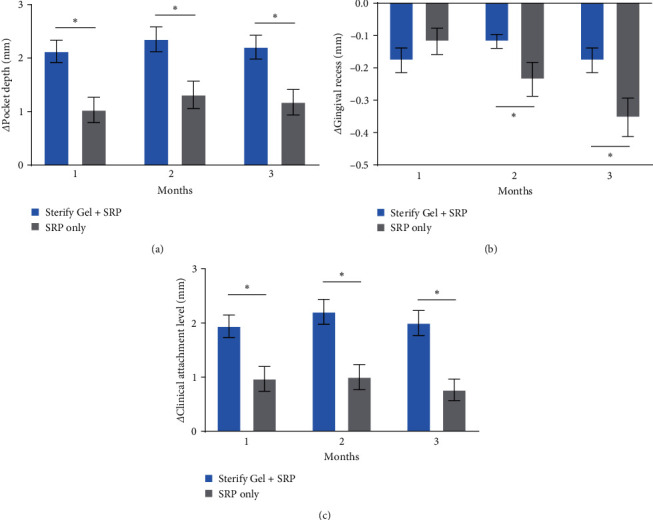
(a) Change in pocket depth (PD) in millimeters at 1, 2, and 3 months vs. pretreatment conditions (*n* = 34). (b) Change in gingival recess in millimeters at 1, 2, and 3 months vs. pretreatment conditions (*n* = 34). (c) Change in clinical attachment level (CAL) in millimeters at 1, 2, and 3 months vs. pretreatment conditions (*n* = 34). Error bars show the standard error of the mean (SEM).  ^*∗*^*p* < 0.05.

Treatment with Sterify Gel in conjunction with SRP consistently demonstrated a noteworthy improvement in PD, surpassing twofold enhancement compared to the control group throughout all follow-up intervals (Figure 1(a)). The mean change in PD compared to the pretreatment condition was 2.06 mm at 1 month, 2.35 mm at 2 months, and 2.21 mm at 3 months in the treatment group; conversely, the mean change in PD compared to the pretreatment condition in the control group was 1.09 mm at 1 month, 1.36 mm at 2 months, and 1.18 mm at 3 months.

Furthermore, minimal gingival recession was observed in all patients. However, patients treated with SRP only had a significant worsening compared to those treated with Sterify Gel at 2 and 3 months (Figure 1(b)). Consistently with PD and gingival recession, significant advancements were also observed in clinical attachment level (CAL) (Figure 1(c)), with the treatment group exhibiting superior progress compared to the control group. Full data on PD, gingival recession, and CAL are available in Table S1.

All group differences in PD, gingival recession, and CAL resulted significant (*p*  < 0.05) when tested with ANOVA. Multiple Wilcoxon tests showed significant differences in all parameters and follow-ups (*p*  < 0.05), with the exception of gingival recess at 1 month (*p*  > 0.05).

### 3.2. Comparison of Bleeding and Plaque Index Scores, Pain Perception, and Adverse Events during Treatment between Sterify Gel and SRP Alone

**Figure 2:**
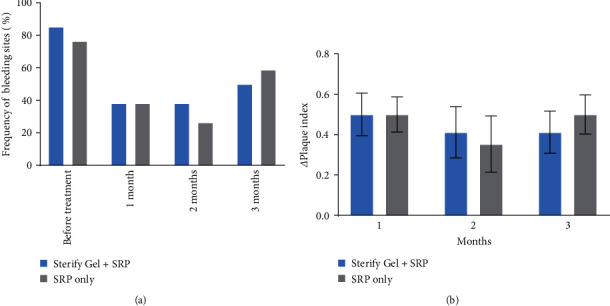
(a) Bleeding index expressed in the frequency of bleeding sites before treatment, at 1, 2, and 3 months (*n* = 34). (b) Change in plaque index at 1, 2, and 3 months vs. pretreatment conditions (*n* = 34). Error bars show SEM.

No significant differences were found in the bleeding (Figure 2(a)) and plaque index (Figure 2(b)) scores between the two groups (*p*  > 0.05). Notably, the bleeding index ameliorated in both groups compared to pretreatment conditions. Similarly, pain perception during the treatment procedure was generally not reported or indicated as absent in most cases (data not shown), resulting in no significant distinction between the Sterify Gel and control groups. Moreover, no adverse events were reported in this study. Full data on plaque index are available in Table S1, and data on bleeding index are available in Table S2.

### 3.3. Influence of Sterify Gel on Tooth Mobility and Furcations

**Figure 3:**
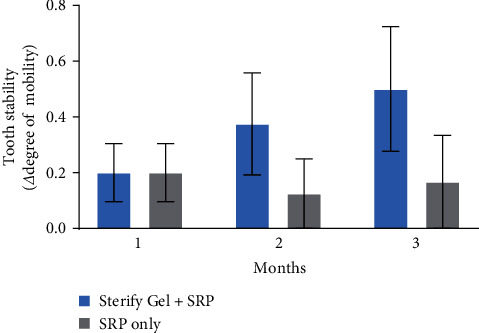
Tooth stability, i.e., change in the degree of mobility compared to before treatment conditions, at 1, 2, and 3 months (*n* = 34). Error bars show SEM.

No significant disparities were observed in tooth mobility (Figure 3) or furcation (data not shown) reduction between the two groups (*p*  > 0.05). Only a minor, although not significant, tendency for better tooth stability was seen at 2 and 3 months in the treatment group compared to the control group. Full data on tooth mobility are available in Table S1.

### 3.4. Reduction of Bacterial Contamination in Periodontal Pockets following Treatment with Sterify Gel

**Figure 4:**
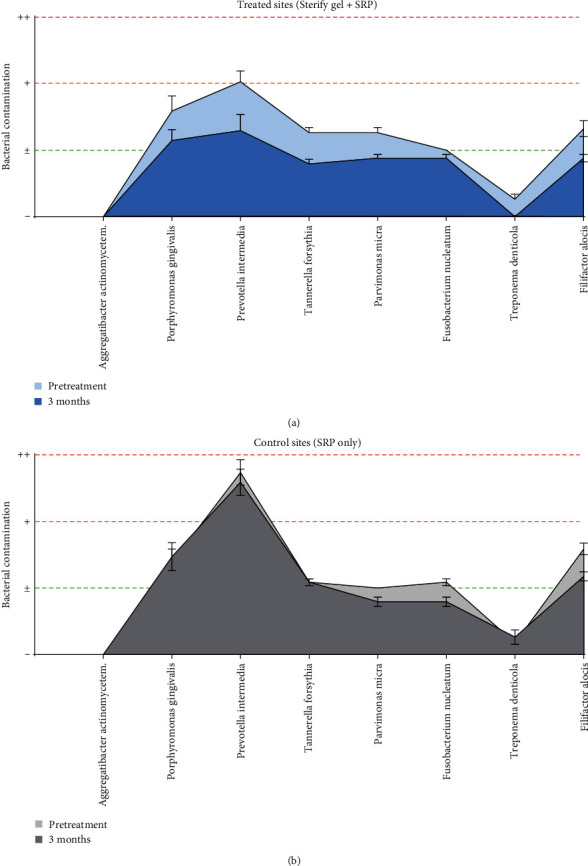
Bacterial contamination of specific bacteria strains was assessed through qPCR of samples obtained with probe collection in (a) treated and (b) control sites. On the *Y*-axis, “−” means negativity (no colony-forming units, CFU); “±” means low positivity (up to 104 CFU); “+” means moderate positivity (between 105 and 106 CFU); and “++” means high positivity (between 106 and 107 CFU). “*Aggregatibacter actinomycetem*” is short for *Aggregatibacter actinomycetemcomitans*. Error bars show SEM.

The frequency of bacterial contamination was comparable between the treatment and control groups at baseline conditions, except for the *Prevotella intermedia* bacterial strain. At 3 months follow-up, a “negative shift” toward less bacterial positivity was observed in the treatment group, indicating a statistically significant trend (*p*  < 0.05) toward reduced bacterial contamination (Figure 4(a)). This trend was not observed in the control group who received SRP only (Figure 4(b)), where no statistically significant differences were found (*p*  > 0.05). Full data on bacterial contamination is available in Table S3.

## 4. Discussion

Our study represents the first clinical investigation of Sterify Gel, an innovative polyvinyl hydrogel, for the treatment of chronic periodontitis. The absence of reported adverse events during the use of Sterify Gel in the study underscores its safety. In addition, our findings demonstrate that treatment with Sterify Gel in conjunction with SRP resulted in significant improvements in periodontal PD, gingival recession, and CAL, compared with SRP alone; while no significant differences were observed in plaque and bleeding index and tooth stability between the two groups. Notably, the “negative shift” toward less bacterial positivity in the treated group suggests the ability of Sterify Gel to protect gingival pockets from bacteria recolonization, which could contribute to the long-term stability of periodontal health. These positive results may be attributed to the mechanical action of occlusion exerted by the hydrogel, which creates a barrier to bacterial recolonization, as well as the antioxidant and bacteriostatic properties of HT and nisin, respectively. Indeed, HT is a naturally occurring antioxidant found in olive oil that garners attention due to its potential health benefits [17, 18]. Acting as a free radical scavenging agent, a tailored concentration of HT can prevent radiation-induced cross-linking and viscosity increase of hydrogels [20], improving hydrogel design and structure.

Similarly, the bacteriostatic properties of nisin [19] further improved the stability of the implanted hydrogel, protecting it from bacterial degradation. HT and nisin may also directly enhance periodontal pocket healing through their properties mentioned above. Moreover, MAP, which is a stable water-soluble form of vitamin C, is used to protect HT from degradation during gamma ray sterilization, acting therefore as a protectant agent in the gel formulation [21].

Using medical devices such as Sterify Gel may represent a promising strategy to overcome the limitations of traditional treatment approaches for chronic periodontitis, such as SRP. While SRP effectively reduces periodontal symptoms, its limited efficacy in deeper and anatomically complex pockets hinders complete bacterial removal, and reinfection may occur rapidly and more severely [6]. The emergence of antimicrobial resistance further highlights the need for new approaches to periodontal disease management, and Sterify Gel may represent a viable alternative to antibiotic-based treatments. Lately, intraperiodontal pocket administration of biomaterials and drugs has emerged as an optimal strategy for the local treatment of periodontitis [22]. In a study evaluating the clinical effects of SRP combined with local administration of hydrogen peroxide gel and doxycycline using customized prescription trays for the treatment of chronic periodontitis, the authors found that the combined treatment resulted in significant reductions in PD compared to SRP alone [23]. Another study aimed to compare the efficacy of subgingivally administered xanthan-based chlorhexidine gel versus 0.2% chlorhexidine irrigation following SRP in the treatment of chronic periodontitis, showing that both groups exhibited statistically significant improvements in clinical parameters compared to SRP alone [24]. Another research group evaluated the adjunctive effect of hyaluronic acid (HA) gel in treating residual periodontal pockets over 12 months with no statistically significant improvement compared to placebo treatment [25]. Our study highlights the potential of Sterify Gel as a novel antibiotic-free adjunct therapy for chronic periodontitis and its role in reducing reliance on antibiotics. Although comparisons with other devices have not yet been made, the innovative formulation of Sterify Gel may overcome the limitations of disinfectants such as chlorhexidine that can cause discoloration of teeth and gum irritation [26].

Limitations of our study include the relatively small sample size, the short follow-up period of 3 months, and the single-center nature, which may limit the generalizability of our findings. Future studies with larger sample sizes, longer follow-up periods, and multicentric framework are necessary to confirm the efficacy of Sterify Gel and explore its potential use in managing periodontal disease. Additionally, future research should further investigate the optimal frequency and duration of Sterify Gel application.

In conclusion, our study demonstrates the potential of Sterify Gel as an effective adjunct therapy for chronic periodontitis. By adhering to the gingival tissue and alveolar bone, Sterify Gel provides comprehensive coverage, supporting the healing process and helping to prevent the recurrence of bacterial infections. Medical devices such as Sterify Gel may represent a viable alternative to antibiotic—and disinfectant-based treatments and address the unmet need for more effective management strategies for periodontal disease. Further research is needed to confirm these findings and explore the broader implications of using medical devices in managing periodontal disease and antimicrobial resistance.

## Data Availability

Data are available upon request due to privacy restrictions.

## Additional Points

*Patents*. Sterify S.r.l. holds patents for its innovative formulations: WO 2021/099981 A1, which covers the controlled-viscosity polymeric hydrogel and its preparation method, and WO 2021/099983 A1, which pertains to the lantibiotic solution against bacterial infections.

## Supplementary Materials

Supplementary Materials The supplementary material accompanying this manuscript comprises additional tables containing a comprehensive compilation of clinical parameters obtained during the clinical trial, including change vs. pretreatment conditions in pocket depth, gingival recess, clinical attachment level, plaque index, degree of mobility. Supplementary tables also describe bleeding index and bacterial contamination in details. Table S1: clinical outcomes measurement between the study groups compared with pretreatment conditions. Table S2: frequency of bleeding sites between the study groups. Table S3: number of observations and frequency of bacterial contamination (all bacteria strains).
