# Supplementary material for: Evaluation of Sterify Gel as an Adjunctive Treatment to Scaling and Root Planing in Promoting Healing of Periodontal Pockets: A Split-Mouth Randomized Controlled Trial
Source: Int J Dent. 2024 Jan 4;2024:3113479. doi: 10.1155/2024/3113479 (PMC10783987; doi:10.1155/2024/3113479)
Supplement: Supplementary Materials — The supplementary material accompanying this manuscript comprises additional tables containing a comprehensive compilation of clinical parameters obtained during the clinical trial, including change vs. pretreatment conditions in pocket depth, gingival recess, clinical attachment level, plaque index, degree of mobility. Supplementary tables also describe bleeding index and bacterial contamination in details. Table S1: clinical outcomes measurement between the study groups compared with pretreatment conditions. Table S2: frequency of bleeding sites between the study groups. Table S3: number of observations and frequency of bacterial contamination (all bacteria strains). [file 3113479.f1.docx]

Evaluation of Sterify Gel as an Adjunctive Treatment to Scaling and Root Planing in Promoting Healing of Periodontal Pockets: A Split-Mouth Randomized Controlled Trial

Luca Levrini ^1^, Michela Rossini ^2,^*, Elisa Truppello ^2^, Simone Sevi ^2^, Enrico Fiorini ^2^, Stefano Benedicenti ^3^, Claudio Pasquale ^3^ and Davide Farronato ^2^

^1^ Department of Human Sciences, Innovation and Territory, postgraduate School of Orthodontic, University of Insubria, 21100 Varese, Italy

^2^ Department of Medicine and Surgery, School of Dental Hygiene, University of Insubria, 21100 Varese, Italy

^3^ Department of Surgical and Diagnostic Sciences, University of Genoa, 16132, Genoa, Italy

***** Correspondence: michela.rossini@uninsubria.it

Supplemental material

| **Change vs.**  **Pre-treatment conditions** | **Sterify Gel + SRP** | | | **SRP only** | | |
| --- | --- | --- | --- | --- | --- | --- |
|  | **1 month** | **2 months** | **3 months** | **1 month** | **2 months** | **3 months** |
| Pocket Depth (mm) | 2.13 ± 1.21 | 2.36 ± 1.35 | 2.21 ± 1.30 | 1.03 ± 1.38 | 1.31 ± 1.50 | 1.18 ± 1.38 |
| Gingival Recess (mm) | -0.18 ± 0.22 | -0.12 ± 0.13 | -0.18 ± 0.22 | -0.12 ± 0.24 | -0.24 ± 0.31 | -0.35 ± 0.35 |
| Clinical Attachment Level (mm) | 1.94 ± 1.20 | 2.20 ± 1.34 | 2.00 ± 1.35 | 0.97 ± 1.34 | 1.00 ± 1.35 | 0.76 ± 0.16 |
|  |  |  |  |  |  |  |
| Plaque Index | 0.50 ± 0.62 | 0.41 ± 0.74 | 0.41 ± 0.61 | 0.50 ± 0.51 | 0.35 ± 0.81 | 0.50 ± 0.56 |
| Degree of Mobility | 0.20 ± 0.41 | 0.38 ± 0.52 | 0.50 ± 0.55 | 0.20 ± 0.41 | 0.13 ± 0.35 | 0.17 ± 0.41 |
|  |  |  |  |  |  |  |

**Table 1**. Clinical outcomes measurement between the study groups compared with pre-treatment conditions. Pocket Depth, Gingival Recess, and Clinical Attachment Level are expressed in millimeters. Plaque Index is expressed as mean values where 0 is no plaque in the gingival area, 1 is a soft film of plaque, 2 is a moderate accumulation of soft deposits, 3 is an abundance of soft matter. Degree of Mobility is expressed as mean values where 0 is physiological mobility, 1 is slightly increased mobility, 2 is significantly increased mobility, 3 is significant mobility with functional impediment.

|  | **Sterify Gel + SRP** | | | | **SRP only** | | | | | | |
| --- | --- | --- | --- | --- | --- | --- | --- | --- | --- | --- | --- |
|  | **Pre-treatment** | **1 month** | **2 months** | **3 months** | **Pre-treatment** | | **1 month** | **2 months** | | **3 months** | |
| Bleeding sites | 85.29% | 38.24% | 38.24% | 50% | 76.47% | 38.23% | | | 26.47% | | 58.82% |
|  |  |  |  |  |  |  | | |  | |  |

**Table 2.** Frequency of bleeding sites between the study groups.

| **Bacterial contamination (number of observations**  **and frequency)** | **Sterify Gel + SRP** | | | **SRP only** | | | | |  |  |
| --- | --- | --- | --- | --- | --- | --- | --- | --- | --- | --- |
|  | **Pre-treatment** | **3 months** | | | **Pre-treatment** | | **3 months** | | |  |
| Class 0 (-) | 69 (26%) | | 105 (39%) | | | 72 (27%) | | 86 (32%) | | |
| Class 1 (+/-) | 136 (50%) | | 152 (56%) | | | 131 (48%) | | 136 (49%) | | |
| Class 2 (+) | 46 (17%) | | 3 (1%) | | | 42 (15%) | | 25 (9%) | | |
| Class 3 (++) | 15 (6%) | | 6 (2%) | | | 15 (6%) | | 20 (7%) | | |
| Class 4 (+++) | 6 (2%) | | 6 (2%) | | | 12 (4%) | | 9 (3%) | | |
|  |  | |  | | |  | |  | | |

**Table 3.** Number of observations and frequency of bacterial contamination (all bacteria strains). “Class 0” or “-“ means negativity (no colony-forming units, CFU); “Class 1” or “+/-“ means low positivity (up to 10^4^ CFU); “Class 2” or “+” means moderate positivity (between 10^5^ and 10^6^ CFU); “Class 3” or “++” means high positivity (between 10^6^ and 10^7^ CFU); “Class 4” or “+++” means severe positivity (above 10^7^ CFU)
